# Supplementary material for: New Insights into the Multivariate Analysis of SER Spectra Collected on Blood Samples for Prostate Cancer Detection: Towards a Better Understanding of the Role Played by Different Biomolecules on Cancer Screening: A Preliminary Study
Source: Cancers (Basel). 2022 Jun 30;14(13):3227. doi: 10.3390/cancers14133227 (PMC9264810; doi:10.3390/cancers14133227)
Supplement: Supplementary file 1 [file cancers-14-03227-s001.zip › cancers-1782241-supplementary.pdf]

cancers MDPI

Supplementary file

New insights into the Multivariate Analysis of SER spectra collected on blood samples for Prostate Cancer Detection: towards a better understanding of the role played by different biomolecules on cancer screening

Vlad Cristian Munteanu, Raluca Andrada Munteanu, Diana Gulei, Radu Mărginean, Vlad Horia Schițcu, Anca Onaciu, Valentin Toma, Gabriela Fabiola Știufiuc, Ioan Coman and Rareș Ionuț Știufiuc

Table S1. The SERS peaks tentative assignments of major vibrational bands in plasma and serum samples from PCa patients and healthy donors.

Peak positions (cm^{-1})	Vibrational mode	Assignments
390	n/a	Uric acid [1]
480	Ring torsion vibration, (N-C-S) stretching [2]	DNA, Guanine, arginine, valine, glutamate, tyrosine, tryptophan, mannose, glucose, acetyl coenzyme A, acetoacetate, cholesterol [2–4]
498	Ring torsion vibration, (N-C-S) and S–S stretching [2,5–7]<br><br>proteins	Protein, lipid or carbohydrate components, guanine, thymine, arginine, valine, glutamate, tyrosine, tryptophan, mannose, glucose, acetyl coenzyme A, acetoacetate, uric acid [1,2,5,7–12]
535	(S-S) stretching in proteins [2,13]	Uracil, alanine, phenylalanine, glutamate, tyrosine, tryptophan, cysteine, mannose, fructose, galactosamine, N-acetyl-D-glucosamine, phosphoenolpyruvate, acetyl coenzyme A, D-fructose-6-phosphate, riboflavin, phosphatidylserine, cholesterol ester [2,5,7]
596	(C-S) and 4(PO_4^{3-}) stretching [2]	Amide VI, ascorbic acid, glutamate, tryptophan, methionine, tyrosine, cytosine, lactose, N-acetyl-D-glucosamine, pyruvate, acetyl coenzyme A, phosphatidylinositol [2,7,14]
642	Ring stretching of uric acid and hypoxanthine [10]<br>(C–S) stretching [5,7,15]<br>(C-C) twisting [8,10,16]<br>Ring breathing modes [17]<br>Skeletal ring deformation [18]	DNA bases, lipids and proteins, tyrosine [5–8,10,12,16,17,19,20]
690	In-phase ring stretching of the six-membered ring except C_4C_5 [11,12,21–23]	Guanine, cytosine, glutathione, tyrosine [1,6,11,12,21–23]
728	Ring stretching in uric acid and hypoxanthine [10]<br>(C-S), (C-C), (C-N), (CH_2), CN^-(CH_3)$_3$, N^+(CH_3)$_3$ stretching [2,17]<br>(C-H) and (N-H) bending [7,10,18]	DNA/RNA bases, glycine, tryptophan, histidine, proline, oleic acid, triolein, mannose, citric acid, coenzyme A, acetyl coenzyme A, glutathione, phospholipids, hypoxanthine, phenylalanine [2,4,21–23,7–13,17]

	Ring breathing modes of DNA/RNA bases and (O-P-O) stretching in DNA [16]	
769	Ring breathing and deformation [2,3]	Cytosine, thymine, uracil, glutamate, tyrosine, tryptophan, fumarate, proline, N-acetyl-D-glucosamine, phosphoenolpyruvate, acetoacetate, phospholipids, riboflavin, pyrimidine, phosphatidylserine [2,3,14]
815	(C–C–O) and (C-C) stretching [5,7,8]<br>Ring vibration [18]	Collagen L-serine, glutathione, uric acid [5,7,8,10]
893	(C-C) stretching [8,19]<br>(C–O–H) and (N-H) bending [2,5,7,8,18]	Deoxyribose phosphate backbone, D-galactosamine, tryptophan, uric acid [5,7,8,10,12,24]
915	($C\text{-}COO^-$) stretching in carbohydrates [25]	Glucose, lactic acid [3]
969	Skeletal vibration [6,24]	Phosphodiester, saccharides, deoxyribose [16]
1010	Symmetric ring breathing mode of phenylalanine [8,15,26]<br>(CO), (CC) and (OCH) bending [3]<br>(C-C) skeletal in phenylalanine [6]	Aromatic aminoacids, carbohydrates, phenylalanine, proteins, lipids, thymine, deoxyribose phosphate backbone, 5-methylcytosine [3,8–10,12,16,23,27,28]
1075	(C-C), (C-N) and (C-O) stretching [2,3,7,8,17]<br>Symmetric stretching of (PO_2^-) [16]<br>(C-S) stretching and (NH_2) rocking [29]	Nucleic acids, saccharides, adenine, glycine, serine, glutamate, tryptophan, histidine, fatty acids, mannose, glucose, lactose, galactosamine, N-acetyl-D-glucosamine, acetyl coenzyme A, D-fructose-6-phosphate, glutathione, phospholipids, collagen, proteins [2,3,7,8,16,17]
1099	(C-N), (C-C) and (PO_2^-) stretching in carbohydrates and lipids [13,16,19,24]	DNA, phenylalanine, proteins, lipids, phospholipids and carbohydrates [12,16,20,22,24]
1136	(C–O), (C-N), (C-C) stretching mode [2,4,5,18,30–33]<br>(C-H), (C-N) bending [4,29,31,32]<br>(CH_3) rocking [4,31,32]	adenine, glycine, serine, glutamate, tryptophan, histidine, fatty acids, mannose, glucose, lactose, galactosamine, N-acetyl-D-glucosamine, acetyl coenzyme A, D-fructose-6-phosphate, glutathione, phospholipids, uric acid, tyrosine, ascorbic acid, D-mannose [2,5,7,8,10,14]
1209	($C\text{-}C_6H_5$) stretching [8,17]<br>(N-C-C) stretching and bending [18]<br>(C_mH) bending [34]	proteins arising from aromatic amino acids such as tyrosine, phenylalanine, tryptophan, adenine, thymine, amide III, uric acid [6–8,10,15–17,19,20]
1256	Ring stretching in uric acid and hypoxanthine [10]<br>(C-N) and (C-C) stretching [3,16]<br>(CH) bending in lipids [13]<br>Asymmetric (PO_2^-) stretching [35]	Proteins, amide III, collagen, lipids, adenine, cytosine [1,3,6,16,17,20,27]
1336	(CH), (CH_2) and (CH_3) bending [2,6,10,19]<br>(CH_3CH_2) wagging in nucleic acids, (CH_2) deformation [16]<br>Symmetric stretch of the (NO_2) group [36]	hydrocarbon chains of the fatty acids, with a possible small contribution from the CH_2 groups of cholesterol, triglycerides, collagen, nucleic acid bases, proteins, tryptophan, valine, serine, arginine, phenylalanine, tyrosine, tryptophan, histidine, proline, lactose, mannose, galactosamine, N-acetyl-D-glucosamine, acetyl coenzyme A, glutathione, phospholipids,

	(C-H) and (CH_3) stretching, (C_3-C_3), (C_5-O_5), (CH_α), (CH_3CH_2), CH deformation, (C-NH_2) stretching in amide II [37]	hypoxanthine, amide III, amide linkages, [2,3,5,7,10,15,16,24,38]
1369	(C-H), (CH_3), (C3-C3), (C5-O5), (CH_α), (CH_3CH_2) stretching [18] (CH_3) and (CH_2) bending, [18] Thymine: C–H + Methyl bend [12,14,28] (COO^-) small stretching [31] Symmetrical stretching of the half ring in pyridine [34]	adenine, thymine, guanine, valine, serine, arginine, phenylalanine, tyrosine, tryptophan, histidine, proline, fatty acids, lactose, mannose, galactosamine, N-acetyl-D-glucosamine, acetyl coenzyme A, glutathione, phospholipids, amid III [2,4,13,16]
1406	(CH_2) deformation [5] (CH_3/CH_2) twisting and bending of lipids and collagen [16] IgG (COO^-) symmetrical stretching [6]	Collagen, lipids and phospholipids, citrate anions. Proteins, uracil [5,6,8,11,16,39]
1447	(CH_2/CH_3) deformation [4,9,34,10,13,15–17,19,24,27]	hydrocarbon chains of the fatty acids, with a possible small contribution from the CH_2 groups of cholesterol, phospholipids, glutathione, deoxyribose [6,14,16,20,38]
1506	n/a	DNA/RNA bases, β-carotene, amide II, tryptophan, phenylalanine [16]
1577	(C=C) bending [5,7] DNA-RNA ring breathing modes of adenine and guanine [6] (C-C) stretching and (NH_2) bend [29,34]	Phenylalanine, DNA/RNA bases [5,6,24,7,8,10–12,16,21,22]
1617	(C=O), (NH_2) and (C=C) stretching [2,9,34]	Adenine, cytosine, thymine, tryptophan, tyrosine, fatty acids, galactosamine, phenylalanine, pyruvate, coenzyme A, acetoacetate, ascorbic acid, amide I, α helix, phospholipids [2,9,16,19,20,40]
1662	(C=O) stretching mode in β-sheets [41]	amide I α-helix conformation, lipids/ proteins [5,8,9,15,16,26,41,42]

Table S2. Different models (PCA-LDA and PLSDA) for both train and test samples.

		PCA-LDA 2	PCA-LDA 5	PCA-LDA 15 (99% EV)	PLSDA 2	PLSDA 5
Plasma	Train	90.1%	89.3%	97.6%	92.7%	100.0%
	Test	92.6%	85.4%	92.7%	90.2%	95.1%
Serum	Train	97.7%	100.0%	100.0%	99.8%	100.0%
	Test	97.7%	100.0%	100.0%	95.3%	100.0%

For both models employing SVMs, we used soft-margin SVMs where the trade-off constant C was selected through a grid search within a nested stratified k-fold cross-validation loop on the training data in each LOOCV fold.

Table S3. The classification results of PCA-SVM, SVM and LDA analyses.

	Sample	AUC	Accuracy	Precision	Sensitivity	Specificity	True Pos.	True Neg.	False Pos.	False Neg.
PCA-SVM (2 PC, Linear)	Plasma	75.0%	82.9%	79.4%	100.0%	50.0%	27	7	7	0
	Serum	92.9%	95.3%	93.5%	100.0%	85.7%	29	13	2	0
SVM (Linear)	Plasma	91.0%	92.7%	92.9%	96.3%	85.7%	26	12	2	1
	Serum	89.3%	93.0%	90.6%	100.0%	78.6%	29	11	3	0
LDA	Plasma	93.9%	95.1%	96.3%	96.3%	92.9%	26	13	1	1
	Serum	100.0%	100.0%	100.0%	100.0%	100.0%	29	14	0	0

We note that the results do not deviate significantly from the ones obtained using the PCA-LDA approach, except for the PCA-SVM case, where performance seems to slightly drop.

The first two principal components in the case of plasma and serum samples are presented in the figures below (Figures S1 and S2).

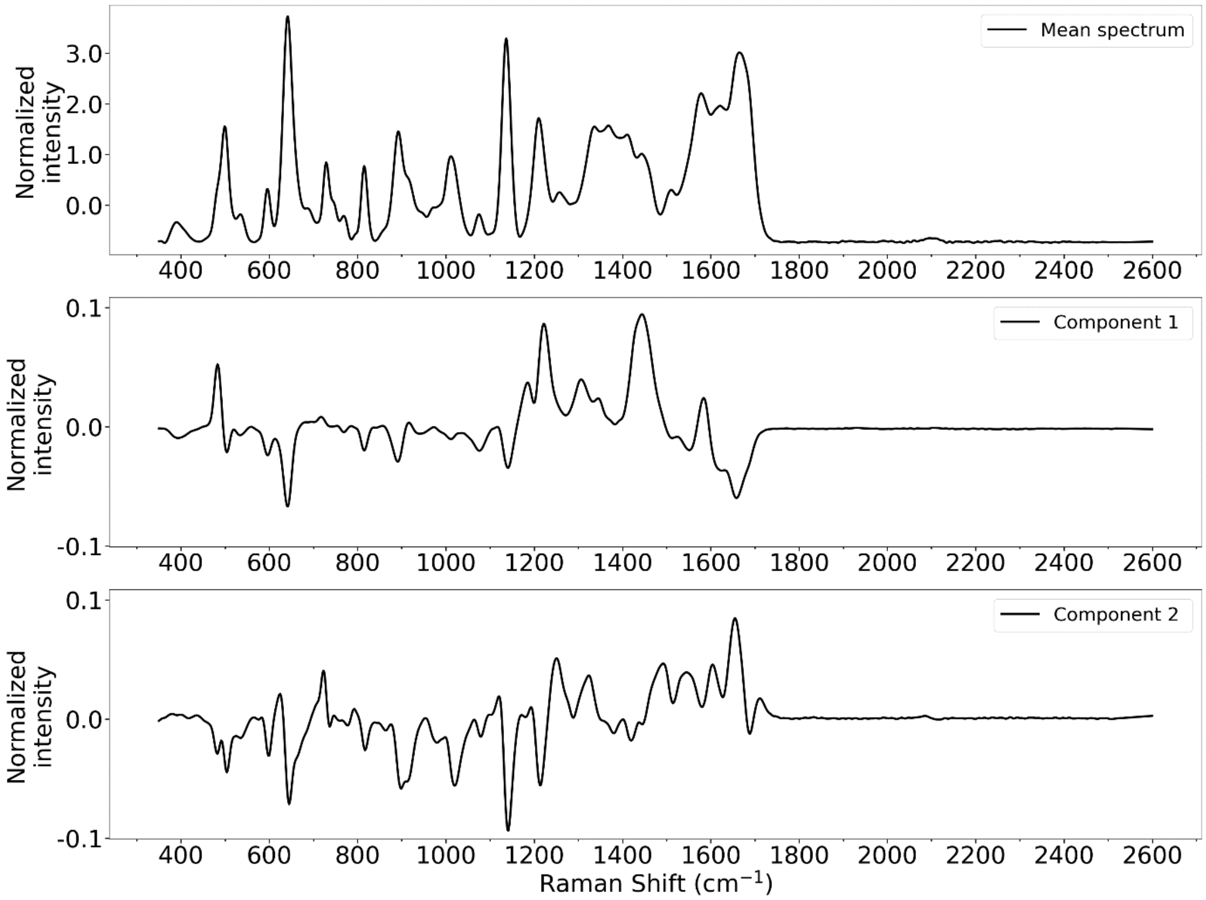

Figure S1. Mean spectrum and first two principal components for plasma spectra.

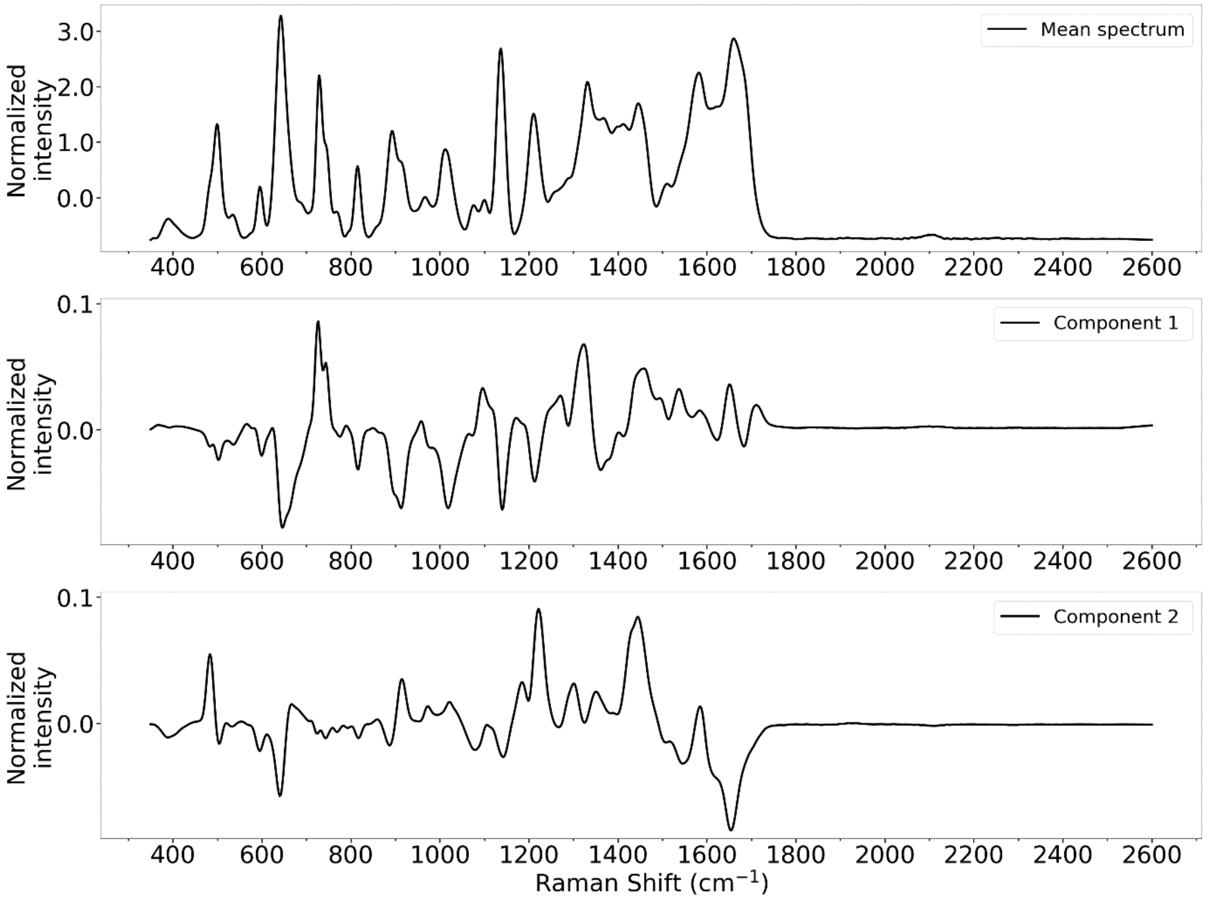

Figure S2. Mean spectrum and first two principal components for serum spectra.

References

1. Tefas, C.; Mărginean, R.; Toma, V.; Petrushev, B.; Fischer, P.; Tanțău, M.; Știufiuc, R. Surface-enhanced Raman scattering for the diagnosis of ulcerative colitis: will it change the rules of the game? *Anal. Bioanal. Chem.* **2021**, *413*, 827–838, doi:10.1007/s00216-020-03036-2.
2. Ryzhikova, E.; Ralbovsky, N.M.; Halámková, L.; Celmins, D.; Malone, P.; Molho, E.; Quinn, J.; Zimmerman, E.A.; Lednev, I.K. Multivariate Statistical Analysis of Surface Enhanced Raman Spectra of Human Serum for Alzheimer's Disease Diagnosis. *Appl. Sci.* **2019**, *9*, 3256, doi:10.3390/app9163256.
3. Gao, N.; Wang, Q.; Tang, J.; Yao, S.; Li, H.; Yue, X.; Fu, J.; Zhong, F.; Wang, T.; Wang, J. Non-invasive SERS serum detection technology combined with multivariate statistical algorithm for simultaneous screening of cervical cancer and breast cancer. *Anal. Bioanal. Chem.* **2021**, *413*, 4775–4784, doi:10.1007/s00216-021-03431-3.
4. Tan, Y.; Yan, B.; Xue, L.; Li, Y.; Luo, X.; Ji, P. Surface-enhanced Raman spectroscopy of blood serum based on gold nanoparticles for the diagnosis of the oral squamous cell carcinoma. *Lipids Health Dis.* **2017**, *16*, 73, doi:10.1186/s12944-017-0465-y.
5. Feng, S.; Lin, D.; Lin, J.; Li, B.; Huang, Z.; Chen, G.; Zhang, W.; Wang, L.; Pan, J.; Chen, R.; et al. Blood plasma surface-enhanced Raman spectroscopy for non-invasive optical detection of cervical cancer. *Analyst* **2013**, *138*, 3967, doi:10.1039/c3an36890d.
6. Bankapur, A.; Zachariah, E.; Chidangil, S.; Valiathan, M.; Mathur, D. Raman Tweezers Spectroscopy of Live, Single Red and White Blood Cells. *PLoS One* **2010**, *5*, e10427, doi:10.1371/journal.pone.0010427.
7. Wu, Q.; Qiu, S.; Yu, Y.; Chen, W.; Lin, H.; Lin, D.; Feng, S.; Chen, R. Assessment of the radiotherapy effect for nasopharyngeal cancer using plasma surface-enhanced Raman spectroscopy technology. *Biomed. Opt. Express* **2018**, *9*, 3413, doi:10.1364/BOE.9.003413.
8. Lin, D.; Pan, J.; Huang, H.; Chen, G.; Qiu, S.; Shi, H.; Chen, W.; Yu, Y.; Feng, S.; Chen, R. Label-free blood plasma test based on surface-enhanced Raman scattering for tumor stages detection in nasopharyngeal cancer. *Sci. Rep.* **2015**, *4*, 4751, doi:10.1038/srep04751.
9. Premasiri, W.R.; Lee, J.C.; Ziegler, L.D. Surface-Enhanced Raman Scattering of Whole Human Blood, Blood Plasma, and Red Blood Cells: Cellular Processes and Bioanalytical Sensing. *J. Phys. Chem. B* **2012**, *116*, 9376–9386, doi:10.1021/jp304932g.
10. Știufiuc, G.F.; Toma, V.; Buse, M.; Mărginean, R.; Morar-Bolba, G.; Culic, B.; Tetean, R.; Leopold, N.; Pavel, I.; Lucaciu, C.M.; et al. Solid Plasmonic Substrates for Breast Cancer Detection by Means of SERS Analysis of Blood Plasma. *Nanomaterials* **2020**, *10*, 1212, doi:10.3390/nano10061212.
11. Otto, C.; van den Tweel, T.J.J.; de Mul, F.F.M.; Greve, J. Surface-enhanced Raman spectroscopy of DNA bases. *J. Raman Spectrosc.* **1986**, *17*, 289–298, doi:10.1002/jrs.1250170311.

12. Prescott, B.; Steinmetz, W.; Thomas, G.J. Characterization of DNA structures by laser Raman spectroscopy. *Biopolymers* **1984**, *23*, 235–256, doi:10.1002/bip.360230206.
13. Xue, L.; Yan, B.; Li, Y.; Tan, Y.; Luo, X.; Wang, M. Surface-enhanced Raman spectroscopy of blood serum based on gold nanoparticles for tumor stages detection and histologic grades classification of oral squamous cell carcinoma. *Int. J. Nanomedicine* **2018**, *Volume 13*, 4977–4986, doi:10.2147/IJN.S167996.
14. De Gelder, J.; De Gussem, K.; Vandenabeele, P.; Moens, L. Reference database of Raman spectra of biological molecules. *J. Raman Spectrosc.* **2007**, *38*, 1133–1147, doi:10.1002/jrs.1734.
15. Auner, G.W.; Koya, S.K.; Huang, C.; Broadbent, B.; Trexler, M.; Auner, Z.; Elias, A.; Mehne, K.C.; Brusatori, M.A. Applications of Raman spectroscopy in cancer diagnosis. *Cancer Metastasis Rev.* **2018**, *37*, 691–717, doi:10.1007/s10555-018-9770-9.
16. Medipally, D.K.R.; Cullen, D.; Untereiner, V.; Sockalingum, G.D.; Maguire, A.; Nguyen, T.N.Q.; Bryant, J.; Noone, E.; Bradshaw, S.; Finn, M.; et al. Vibrational spectroscopy of liquid biopsies for prostate cancer diagnosis. *Ther. Adv. Med. Oncol.* **2020**, *12*, 175883592091849, doi:10.1177/1758835920918499.
17. Cao, X.; Wang, Z.; Bi, L.; Zheng, J. Label-Free Detection of Human Serum Using Surface-Enhanced Raman Spectroscopy Based on Highly Branched Gold Nanoparticle Substrates for Discrimination of Non-Small Cell Lung Cancer. *J. Chem.* **2018**, *2018*, 1–13, doi:10.1155/2018/9012645.
18. Westley, C.; Xu, Y.; Thilaganathan, B.; Carnell, A.J.; Turner, N.J.; Goodacre, R. Absolute Quantification of Uric Acid in Human Urine Using Surface Enhanced Raman Scattering with the Standard Addition Method. *Anal. Chem.* **2017**, *89*, 2472–2477, doi:10.1021/acs.analchem.6b04588.
19. Dingari, N.C.; Horowitz, G.L.; Kang, J.W.; Dasari, R.R.; Barman, I. Raman Spectroscopy Provides a Powerful Diagnostic Tool for Accurate Determination of Albumin Glycation. *PLoS One* **2012**, *7*, e32406, doi:10.1371/journal.pone.0032406.
20. González-Solís, J. Discrimination of different cancer types clustering Raman spectra by a super paramagnetic stochastic network approach. *PLoS One* **2019**, *14*, e0213621, doi:10.1371/journal.pone.0213621.
21. Garcia-Rico, E.; Alvarez-Puebla, R.A.; Guerrini, L. Direct surface-enhanced Raman scattering (SERS) spectroscopy of nucleic acids: from fundamental studies to real-life applications. *Chem. Soc. Rev.* **2018**, *47*, 4909–4923, doi:10.1039/C7CS00809K.
22. Barhoumi, A.; Zhang, D.; Tam, F.; Halas, N.J. Surface-Enhanced Raman Spectroscopy of DNA. *J. Am. Chem. Soc.* **2008**, *130*, 5523–5529, doi:10.1021/ja800023j.
23. Moisoiu, V.; Stefancu, A.; Iancu, S.D.; Moisoiu, T.; Loga, L.; Dican, L.; Alecsa, C.D.; Boros, I.; Jurj, A.; Dima, D.; et al. SERS assessment of the cancer-specific methylation pattern of genomic DNA: towards the detection of acute myeloid leukemia in patients undergoing hematopoietic stem cell transplantation. *Anal. Bioanal. Chem.* **2019**, *411*, 7907–7913, doi:10.1007/s00216-019-02213-2.
24. Schneider, F.W. Frank S. Parker: Applications of Infrared, Raman, and Resonance Raman Spectroscopy in Biochemistry , Plenum Press, New York and London 1983. 550 Seiten, Preis: $ 78,-. *Berichte der Bunsengesellschaft für Phys. Chemie* **1984**, *88*, 1167B – 1168, doi:10.1002/bbpc.198400034.
25. Fan, C.; Hu, Z.; Mustapha, A.; Lin, M. Rapid detection of food- and waterborne bacteria using surface-enhanced Raman spectroscopy coupled with silver nanosubstrates. *Appl. Microbiol. Biotechnol.* **2011**, *92*, 1053–1061, doi:10.1007/s00253-011-3634-3.
26. Stefancu, A.; Moisoiu, V.; Couti, R.; Andras, I.; Rahota, R.; Crisan, D.; Pavel, I.E.; Socaciu, C.; Leopold, N.; Crisan, N. Combining SERS analysis of serum with PSA levels for improving the detection of prostate cancer. *Nanomedicine* **2018**, *13*, 2455–2467, doi:10.2217/nnm-2018-0127.
27. Bonifacio, A.; Dalla Marta, S.; Spizzo, R.; Cervo, S.; Steffan, A.; Colombatti, A.; Sergo, V. Surface-enhanced Raman spectroscopy of blood plasma and serum using Ag and Au nanoparticles: a systematic study. *Anal. Bioanal. Chem.* **2014**, *406*, 2355–2365, doi:10.1007/s00216-014-7622-1.
28. Aroca, R.; Bujalski, R. Surface enhanced vibrational spectra of thymine. *Vib. Spectrosc.* **1999**, *19*, 11–21, doi:10.1016/S0924-2031(99)00003-X.
29. Domenici, F.; Bizzarri, A.R.; Cannistraro, S. Surface-enhanced Raman scattering detection of wild-type and mutant p53 proteins at very low concentration in human serum. *Anal. Biochem.* **2012**, *421*, 9–15, doi:10.1016/j.ab.2011.10.010.
30. Xiaoming, D.; Yoshinori, Y.; Hiroshi, Y.; Harumi, U.; Ozaki, Y. Biological Applications of Anti-Stokes Raman Spectroscopy: Quantitative Analysis of Glucose in Plasma and Serum by a Highly Sensitive Multichannel Raman Spectrometer. *Appl. Spectrosc.* **1996**, *50*, 1301–1306.
31. Kamińska, A.; Winkler, K.; Kowalska, A.; Witkowska, E.; Szymborski, T.; Janeczek, A.; Waluk, J. SERS-based Immunoassay in a Microfluidic System for the Multiplexed Recognition of Interleukins from Blood Plasma: Towards Picogram Detection. *Sci. Rep.* **2017**, *7*, 10656, doi:10.1038/s41598-017-11152-w.
32. Grubisha, D.S.; Lipert, R.J.; Park, H.-Y.; Driskell, J.; Porter, M.D. Femtomolar Detection of Prostate-Specific Antigen: An Immunoassay Based on Surface-Enhanced Raman Scattering and Immunogold Labels. *Anal. Chem.* **2003**, *75*, 5936–5943, doi:10.1021/ac034356f.
33. Jarvis, R.M.; Brooker, A.; Goodacre, R. Surface-Enhanced Raman Spectroscopy for Bacterial Discrimination Utilizing a Scanning Electron Microscope with a Raman Spectroscopy Interface. *Anal. Chem.* **2004**, *76*, 5198–5202, doi:10.1021/ac049663f.
34. Atkins, C.G.; Buckley, K.; Blades, M.W.; Turner, R.F.B. Raman Spectroscopy of Blood and Blood Components. *Appl. Spectrosc.* **2017**, *71*, 767–793, doi:10.1177/0003702816686593.
35. Xie, Y.; Xu, L.; Wang, Y.; Shao, J.; Wang, L.; Wang, H.; Qian, H.; Yao, W. Label-free detection of the foodborne pathogens of Enterobacteriaceae by surface-enhanced Raman spectroscopy. *Anal. Methods* **2013**, *5*, 946–952, doi:10.1039/C2AY26107C.

36. Wang, G.; Lipert, R.J.; Jain, M.; Kaur, S.; Chakraboty, S.; Torres, M.P.; Batra, S.K.; Brand, R.E.; Porter, M.D. Detection of the potential pancreatic cancer marker MUC4 in serum using surface-enhanced Raman scattering. *Anal. Chem.* **2011**, *83*, 2554–61, doi:10.1021/ac102829b.
37. Maquelin, K.; Choo-Smith, L.-P.; van Vreeswijk, T.; Endtz, H.P.; Smith, B.; Bennett, R.; Bruining, H.A.; Puppels, G.J. Raman Spectroscopic Method for Identification of Clinically Relevant Microorganisms Growing on Solid Culture Medium. *Anal. Chem.* **2000**, *72*, 12–19, doi:10.1021/ac991011h.
38. Bulkin, B.J. Raman spectroscopic study of human erythrocyte membranes. *Biochim. Biophys. Acta - Biomembr.* **1972**, *274*, 649–651, doi:10.1016/0005-2736(72)90214-3.
39. Munro, C.H.; Smith, W.E.; Garner, M.; Clarkson, J.; White, P.C. Characterization of the Surface of a Citrate-Reduced Colloid Optimized for Use as a Substrate for Surface-Enhanced Resonance Raman Scattering. *Langmuir* **1995**, *11*, 3712–3720, doi:10.1021/la00010a021.
40. Schuster, K.C.; Urlaub, E.; Gapes, J.R. Single-cell analysis of bacteria by Raman microscopy: spectral information on the chemical composition of cells and on the heterogeneity in a culture. *J. Microbiol. Methods* **2000**, *42*, 29–38, doi:10.1016/S0167-7012(00)00169-X.
41. Maiti, N.C.; Apetri, M.M.; Zagorski, M.G.; Carey, P.R.; Anderson, V.E. Raman Spectroscopic Characterization of Secondary Structure in Natively Unfolded Proteins: α-Synuclein. *J. Am. Chem. Soc.* **2004**, *126*, 2399–2408, doi:10.1021/ja0356176.
42. Huefner, A.; Kuan, W.-L.; Mason, S.L.; Mahajan, S.; Barker, R.A. Serum Raman spectroscopy as a diagnostic tool in patients with Huntington's disease. *Chem. Sci.* **2020**, *11*, 525–533, doi:10.1039/C9SC03711J.
